# Pathogenesis and Treatment of Neurologic Diseases Associated With *Mycoplasma pneumoniae* Infection

**DOI:** 10.3389/fmicb.2018.02751

**Published:** 2018-11-20

**Authors:** Renato D’Alonzo, Elisabetta Mencaroni, Lorenza Di Genova, Daniela Laino, Nicola Principi, Susanna Esposito

**Affiliations:** ^1^Pediatric Clinic, Department of Surgical and Biomedical Sciences, Università degli Studi di Perugia, Perugia, Italy; ^2^Università degli Studi di Milano, Milan, Italy

**Keywords:** antinfective therapy, central nervous system, *Mycoplasma pneumoniae*, neuropathies, peripheral nervous system

## Abstract

*Mycoplasma pneumoniae* is mainly recognized as a respiratory pathogen, although it is associated with the development of several extra-respiratory conditions in up to 25% of the cases. Diseases affecting the nervous system, both the peripheral (PNS) and the central nervous system (CNS), are the most severe. In some cases, particularly those that involve the CNS, *M. pneumoniae*-related neuropathies can lead to death or to persistent neurologic problems with a significant impact on health and a non-marginal reduction in the quality of life of the patients. However, the pathogenesis of most of the *M. pneumoniae*-related neuropathies remains undefined. The main aim of this paper is to discuss what is presently known regarding the pathogenesis and treatment of the most common neurologic disorders associated with *M. pneumoniae* infection. Unfortunately, the lack of knowledge of the true pathogenesis of most of the cases of *M. pneumoniae*-mediated neurological diseases explains why treatment is not precisely defined. However, antibiotic treatment with drugs that are active against *M. pneumoniae* and able to pass the blood-brain barrier is recommended, even though the best drug, dosage, and duration of therapy have not been established. Sporadic clinical reports seem to indicate that because immunity plays a relevant role in the severity of the condition and outcome, attempts to reduce the immune response can be useful. However, further studies are needed before the problem of the best therapy for *M. pneumoniae*-mediated neurological diseases can be efficiently solved.

## Introduction

*Mycoplasma pneumoniae* is a cell-wall deficient organism that extracellularly infects the respiratory tract as filamentous forms that adhere to respiratory epithelial cells (Principi and Esposito, 2001). It is transmitted by the respiratory route, and the incubation period from infection to disease is roughly 2–4 weeks. It is ubiquitous, active throughout the year, and there is evidence that it causes endemic infections with periodic epidemics at 4–7 year intervals (Principi and Esposito, 2001). Outbreaks can happen in institutional settings such as schools and summer camps.

*Mycoplasma pneumoniae* is mainly recognized as a respiratory pathogen. It is one of the major causes of both upper and lower respiratory tract infection in children and adults. More than 20% of non-streptococcal pharyngitis cases are due to *M. pneumoniae*, with an even higher incidence among children with recurrent episodes (Esposito et al., 2004). Up to 40% of cases of community-acquired pneumonia (CAP) are associated with a diagnosis of *M. pneumoniae* infection, both in children and adults (Vergis and Yu, 1997; Woodhead, 1998; Ruiz-González et al., 1999; Principi et al., 2001; Baer et al., 2003; Michelow et al., 2004). Moreover, some patients with *M. pneumoniae* infection experience bronco-obstructive signs and symptoms, and *M. pneumoniae* is presently considered a trigger for asthma exacerbations (Esposito et al., 2000; Watanabe et al., 2014).

However, in addition to respiratory diseases, *M. pneumoniae* is associated with the development of several extra-respiratory conditions in a great number of cases without a previous, clinically evident respiratory episode (Narita, 2016). Up to 25% of *M. pneumoniae* respiratory infections have reportedly been complicated by the involvement of various extra-respiratory sites (de Groot et al., 2017). According to the most recent observations (Narita, 2016), extra-respiratory manifestations of *M. pneumoniae* infection include diseases of the skin, the urogenital tract, and some sensory and digestive organs, as well as the cardiovascular, haematopoietic, musculoskeletal and nervous systems. Diseases due to *M. pneumoniae* affecting the nervous system, both the peripheral (PNS) and the central nervous system (CNS), are the most difficult to be diagnosed and treated, and represent a real medical emergency. They are reported in 5% of hospitalized patients whereas *M. pneumoniae* positivity can be detected in 5–10% of patients presenting with acute, febrile CNS disease (Lind et al., 1979; Ponka, 1980; Yiş et al., 2008; Pillai et al., 2015). In some cases, particularly those that involve the CNS, *M. pneumoniae*-related neuropathies can lead to death or to persistent neurologic problems with a significant impact on health and a non-marginal reduction in the quality of life of the patients (Kammer et al., 2016; Narita, 2016). Several studies have tried to understand how *M. pneumoniae* can affect the nervous system and why different clinical manifestations of neurological damage can occur (Waites and Talkington, 2004; Narita, 2010; de Groot et al., 2017). However, the results are disappointing, and the pathogenesis of most of the *M. pneumoniae*-related neuropathies remains undefined. The inability to establish a direct relationship between a previous *M. pneumoniae* infection and the development of neurological signs and symptoms is the main reason for this limitation. As previously reported, neurological impairment frequently occurs without the respiratory symptoms that can lead clinicians to consider and diagnose a *M. pneumoniae* infection. Moreover, when present, respiratory findings are not specific. Finally, laboratory confirmation of *M. pneumoniae* infection is difficult. Its culture is complicated and slow, and serologic tests, which are considered to be the gold standard for the diagnosis of *M. pneumoniae* infection, are only really effective in the identification of *M. pneumoniae* cases when both acute-phase and convalescent-phase serum specimens are available. This limitation makes them useless for the etiological diagnosis of acute diseases, unless retrospectively. The combined use of acute-phase serology and molecular biology tests able to identify *M. pneumoniae* DNA has been suggested (Qu et al., 2013). Unfortunately, evidence of *M. pneumoniae* DNA in upper respiratory secretions in some cases can represent a carrier state as some healthy children especially during outbreaks of *M. infection* were found to carry the pathogen in the nasopharynx (Spuesens et al., 2013). Definitive diagnosis can be made when DNA is detected in the nervous tissue or in the cerebrospinal fluid (CSF). However, in addition to the difficulty of obtaining adequate samples for analysis, *M. pneumoniae* DNA can be undetectable even in cases suggesting *M. pneumoniae* CNS disease (Narita et al., 1992).

For the reasons reported above, the pathogenesis of *M. pneumoniae*-related neuropathies remains frequently unknown. This has important consequences for the treatment of these forms, which remains, even today, based on what has been established for neuropathies with similar symptoms but not strictly linked to *M. pneumoniae* infection. The main aim of this paper is to discuss what is presently known regarding the pathogenesis and treatment of the most common neurologic disorders associated with *M. pneumoniae* infection. The following keywords were used to search among the world medical library collections (Medline, Embase, Cochrane and Cinahl): “*Mycoplasma pneumoniae*” and “peripheral nervous system” or “CNS” or “neuropathy.” We covered the period between January 1, 2007, and December 31, 2017. A total of 267 papers were analyzed. Only papers written in English were considered.

## Hypothesized Pathogeneses and Clinical Manifestations of *Mycoplasma pneumoniae* Neurologic Disorders

The neurological manifestations of *M. pneumoniae* infections have been thought to derive from three different mechanisms. A direct type in which damage of nervous tissue is strictly related to the local activity of *M. pneumoniae*, an indirect type mainly based on autoimmunity, and a vascular type in which local vasculitis or thrombotic vascular occlusion occur through a direct or indirect mechanism (Narita, 2010). The mechanisms are not mutually exclusive, explaining why, in some patients, lesions related to different pathogeneses can be detected.

### Direct Type of Nervous System Damage

*Mycoplasma pneumoniae* infecting the respiratory tract can be transferred to the CNS through gaps between epithelial respiratory cells and cause direct structural and functional lesions in different organs and body systems, including the CNS (Narita et al., 1996). As extra-respiratory manifestations of *M. pneumoniae* infection have been described more commonly in children than in adults (Narita, 2010) and have been repeatedly described in immunocompromised patients (Taylor-Robinson et al., 1978; O’Sullivan et al., 2004), *M. pneumoniae* transfer to the CNS was supposed to be more frequent in subjects with a partially defective immune system (Narita, 2010). However, transfer to the CNS has been substantiated by the detection of *M. pneumoniae* DNA in the CSF or in the blood of some, even if not all, the patients with a clinically and serologically (i.e., by the presence of antibodies against *M. pneumoniae* in serum) confirmed *M. pneumoniae* infection. The genome of this atypical pathogen was detected in CSF in 1 of the 25 patients with pneumonia and 10 of the 17 patients without pneumonia (Narita et al., 1996; Narita, 2000).

When the pathogen reaches the CNS, it can cause damage directly. Generally, it is hypothesized that this damage occurs within 7 days of the respiratory infection, explaining why CNS diseases of the direct type are defined as early-onset diseases. The ability of *M. pneumoniae* to immediately damage host cells is evidenced by experimental studies that have measured the impact of *M. pneumoniae* infection on the epithelial cells of the respiratory tract. *M. pneumoniae* has been shown to adhere to glycoproteins and glycolipids of target cells through the activity of a number of proteins contained in an organelle located at the pole of the pathogen (Chaudhry et al., 2007). P1 adhesin is the main factor responsible for binding, as shown by the evidence that genetically modified *M. pneumoniae* strains lacking this adhesion factor cannot cause infection on a variety of human cells in culture (Kahane, 1984). Moreover, this protein, together with other adhesins (P30, P40 and P90), confers sliding motility to *M. pneumoniae*, thereby favoring the infectious properties of *M. pneumoniae*. Other proteins (HMW-1, HMW-2, HMW-3 and proteins A, B, and C), although not adhesins, seem essential for *M. pneumoniae* cell attachment (Chaudhry et al., 2016). Interestingly, whereas the immunodominant epitopes of the P1 protein are highly variable, the adherence-mediating domains are conserved in HEp-2 cells lines (Chourasia et al., 2014).

As the host immune response is mainly against immunodominant epitopes (Kim et al., 2011), host defenses are partially ineffective in reducing or eliminating pathogen adhesions. Following adhesion, *M. pneumoniae* damages respiratory cells through the generation of hydrogen peroxide and superoxide radicals, which cause oxidative stress and lead to the structural and functional deterioration of cilia with decreased alveolar fluid clearance (Waites and Talkington, 2004). Damage is also produced by a cytotoxin named community-acquired respiratory distress syndrome toxin that has been found capable of inducing vacuolization, karyopyknosis, and loss of epithelial integrity (Kannan and Baseman, 2006; Hardy et al., 2009). This mechanism could have a role also in CNS diseases due to *M. pneumoniae*.

Finally, some studies showed that *M. pneumoniae* harbors a large number of lipoprotein genes, most of which are of unknown function (Hallamaa et al., 2006, 2008). Because of their location on the cell surface, these proteins are likely to be involved in the bacterial response to environmental changes, or in the initial stages of infection. In particular, some authors showed that several lipoproteins of *M. pneumoniae* (MALP2, P48, M161Ag, N-ALP1/NALP2, and F0F1-ATPase) seem to stimulate the host immune system via Toll-like receptors (TLRs), mainly TLR1, TLR2, and TLR6 (Andrews et al., 2013). This stimulation could promote the production of cytokines and chemokines and the development of the humoural and cellular immune response, leading to the histological findings seen in human *M. pneumoniae* CAP (Miyashita et al., 2017). Similar pathogenetic mechanisms can be supposed for early-onset CNS *M. pneumoniae* diseases, as increased cytokine and chemokine concentrations have been found in patients with these diseases (Narita et al., 2005). Narita et al. (2005) examined interleukin (IL)-6, IL-8, IL-18, interferon (INF)-γ, tumour necrosis factor (TNF)-α, and transforming growth factor (TGF)-beta 1 in serum and CFS samples from patients with several CNS manifestations during acute *M. pneumoniae* infection. A significant increase in the concentrations of IL-6 and IL-8 in the CSF of children with early-onset encephalitis and aseptic meningitis was found. Unfortunately, *M. pneumoniae* DNA and cytokines cannot be detected in the CSF of all cases of early-onset disease, highlighting the difficulty of the identification of the true pathogenetic mechanisms of a *M. pneumoniae* -related neuropathy.

### Indirect Type of Nervous System Damage

Some patients suffer from a neurologic disease several days after the onset of a *M. pneumoniae* respiratory episode. When the time passed from the initial *M. pneumoniae* infection is ≥8 days, neurologic manifestations are considered to be a late-onset disease. In these conditions, unlike what can occur in several early-onset diseases, *M. pneumoniae* DNA is only exceptionally detected in the CSF (Narita et al., 1995; Meyer Sauteur et al., 2014). In contrast, in a relevant number of cases, antibodies to *M. pneumoniae* can be found in the CSF and/or in the serum, suggesting that late-onset diseases are mainly based on autoimmunity (Barbagallo et al., 2017). Molecular mimicry between some *M. pneumoniae* components and host myelin glycolipids is considered the pathomechanism of this immune-mediated process (Kusunoki et al., 2001). In particular, *M. pneumoniae* P1 adhesin and glycolipids of the pathogen form a galactocerebroside C (GalC)-like structure that elicit cross-reactive antibody responses against myelin components of the host, i.e., GalC and gangliosides that cross-react with GalCs such as GQ1B (Kuwahara et al., 2017).

The relevance of *M. pneumoniae* components as antigens is supported by the evidence that, in experimental animal models, sensitization with GalC causes demyelinating polyneuropathy (Saida et al., 1981). Moreover, detection of antibodies against GalC and gangliosides in patients with PNS diseases is relatively common (Sauteur et al., 2015; Meyer Sauteur et al., 2016a; Kuwahara et al., 2017). Anti-*M. pneumoniae* antibodies that cross-react with myelin components of the host can be detected in both the serum and in the CSF of patients with *M. pneumoniae*-related neuropathies. However, when they are only detected in the serum, their importance in the acute phase of these diseases is debated as they can be a marker of a previous infection occurred in absence of nervous system involvement (Nishimura et al., 1996; Kikuchi et al., 1997; Kumada et al., 1997; Ang et al., 2002; Susuki et al., 2004; Christie et al., 2007a; Sugeno et al., 2012). In contrast, detection of anti-*M. pneumoniae* antibodies in the CSF, particularly without evidence of *M. pneumoniae* DNA, seems to be more reliable evidence of the indirect pathogenesis of a number of CNS diseases. In these conditions, antibodies can be produced intrathecally, as evidenced by the higher level of antibodies detected in the CSF than in the serum, cross the blood-brain barrier, or reach the CNS through the blood-brain barrier due to increased permeability (Jacobs et al., 1995; Christie et al., 2007a). In this case, increased blood-brain barrier permeability can be hypothesized to depend on cytokine inflammation mediated by the direct effect of *M. pneumoniae*, suggesting a combined pathogenetic mechanism.

### Vascular Type of Nervous System Damage

Neurologic syndromes of the vascular type have been described as disorders of vascular origin involving both direct and indirect mechanisms. Therefore, *M. pneumoniae* may locally induce cytokines and chemokines, such as TNF-α and IL-8, which cause local vasculitic or thrombotic vascular occlusion without a systemic hypercoagulable state (direct type). Alternatively, a generalized thrombotic vascular occlusion may occur as a result of a systemic hypercoagulable state, which is related to an immune-mediated activation of chemical mediators such as fibrin-D-dimer and activated complements (indirect type) (Narita, 2010).

## Relationship Between Suggested Pathogenetic Mechanisms and the Most Common Clinical Manifestations

As previously evidenced, the pathogenetic mechanisms of neurologic *M. pneumoniae*-mediated diseases are not mutually exclusive. This overlap explains why it is very difficult to attribute a single pathogenetic mechanism to each of the various *M. pneumoniae*-related neurological diseases. An exception is made for aseptic meningitis, for which available evidence seems to indicate that the disease is directly caused by *M. pneumoniae*. In several cases, CSF analysis has led to the identification of *M. pneumoniae* DNA and to increased IL-6 and IL-8 concentrations. Moreover, no *M. pneumoniae* antigens have ever been detected in the CNS (Narita et al., 1995; Socan et al., 2001).

Encephalitis and transverse myelitis can be due to both direct and indirect pathogeneses. *M. pneumoniae* encephalitis accounts for 5–30% of all of the reported encephalitis cases, about two-thirds of which occur in children (Christie et al., 2007b). *M. pneumoniae* encephalitis seems to be more commonly due to an indirect mechanism as shown by the low number of cases with positive DNA detection in the CSF. In the United States, among 111 patients with *M. pneumoniae* encephalitis, *M. pneumoniae* DNA was only detected in two cases (Christie et al., 2007a). The low prevalence of *M. pneumoniae* DNA in the CSF has also been reported in studies carried out in Suisse (Meyer Sauteur et al., 2016b), France (Mailles et al., 2009), the United Kingdom (Granerod et al., 2010), and Canada (Bitnun et al., 2001).

Similar conclusions can be drawn for transverse myelitis (Abele-Horn et al., 1998). In a study in which neurological complications of polymerase chain reaction (PCR)-proven *M. pneumoniae* infection in children were evaluated, 12% of the 365 enrolled children had definitive, probable or possible transverse myelitis, but most had prodrome symptoms ≥7 days and serologic evidence of antibodies against *M. pneumoniae* but no presence of *M. pneumoniae* DNA in the CSF (Al-Zaidy et al., 2015). Cerebellitis, opsoclonus-myoclonus syndrome, cranial/peripheral neuropathies, and acute disseminated encephalomyelitis (ADEM) are mainly considered to be due to an indirect pathogenesis, although in some cases, data suggesting a possible direct pathogenesis have been collected. Cerebellitis has been described to be a part of encephalitis and an isolated condition. An indirect pathogenesis was mainly suggested by the long latency between respiratory and neurological symptoms (Schmucker et al., 2014). However, in a case, nervous tissue infiltration with macrophages and lymphocytes, typical of directly determined cases, seemed to indicate a possible pathogenetic alternative (Simpkins et al., 2012). ADEM is known as an immune complex-mediated vasculopathy in which circulating immune complexes are deposited in small venules in the CNS, leading to activation of the serum complement system. An immune-mediated pathogenesis seemed true also for cases secondary to *M. pneumoniae* infection, as suggested by the long latency between infection and brain damage development and the evidence that treatment with anti-inflammatory and immunosuppressive drugs together with plasma exchange can improve the clinical course of the disease (Gupta et al., 2009). However, doubts are raised by the detection of *M. pneumoniae* DNA in the CSF of a patient with *M. pneumoniae*-related ADEM (Matsumoto et al., 2009).

The pathogenesis of mild encephalitis/encephalopathy with a reversible splenial lesion (MERS) is undefined. This is a recently described clinical-radiological syndrome in which neurological dysfunction, including delirious behavior, consciousness disturbance and seizures, is reported in association with damage of the splenium of the corpus callosum alone or in conjunction with the adjacent white matter (Ueda et al., 2016; Yuan et al., 2016). MERS is classified as an autoimmune disease because *M. pneumoniae* DNA is usually not detected in the CSF. However, in a recent review of the described cases, MERS was classified as an early-onset disease (Ueda et al., 2016). Moreover, in one case, increased IL-6 concentrations were found (Yuan et al., 2016). Both of these findings suggest a direct pathogenesis.

GBS is the prototype of autoimmune disorders that follow an infection, and *M. pneumoniae* is considered among the most common causes of this disease. Approximately 15% of GBS cases are associated with a previous *M. pneumoniae* infection, placing this infectious agent in second place after *Campylobacter jejuni* in the list of the most common infectious causes of GBS (Sinha et al., 2007; Esposito and Longo, 2017). However, the role of *M. pneumoniae* in GBS is debated, mainly because antibodies against GalC and gangliosides are detected in only some but not all cases of the disease and can also be observed in healthy controls, although at a lower rate. In a study, serum anti- *M. pneumoniae* antibodies were detected in 3% of adults and 21% of children with GBS and in 0 and 7% of healthy adults and children, respectively (Meyer Sauteur et al., 2016a). Serum anti-GalC antibodies were found in 4% of adults and 25% of children with GBS and in none of controls. The CSF detection of anti-GalC antibodies supports autoimmune pathogenesis, even in rare cases of GBS with CNS involvement. However, a direct mechanism is suggested by the report of a case with *M. pneumoniae* DNA in the CSF without any antibody detection (Meyer Sauteur et al., 2014).

Regarding cases of vasculitis or vasculopathy among the *M. pneumoniae*-associated CNS pathological conditions, stroke and striatal necrosis are those most commonly studied. Stroke can occur in both children and adults, although rarely (Socan et al., 2001; Leonardi et al., 2005). Early presentation was common, and in some of these cases, the pathogen was detected in the CSF, suggesting a direct effect (Padovan et al., 2001). Bilateral striatal necrosis, which resembles a vascular CNS disease called acute necrotizing encephalopathy, is thought to be due to vascular damage. Also, in this case, *M. pneumoniae* damage seems to be of the direct type as the pathogen was found in the CSF of a patient with this disease (Esposito and Longo, 2017).

## Treatment

The proper treatment of neurologic manifestations of *M. pneumoniae* infection is debated. Most of the authors agree with the idea that an antibiotic should be systematically administered regardless of the type of disease and its supposed pathogenesis, but the choice of the best drug to prescribe in each patient is frequently discordant (Principi and Esposito, 1999). Moreover, poor agreement also exists on the need to administer immunoglobulins and/or immunosuppressive drugs in order to limit autoimmunity-related damage.

### Antibiotic Administration

Available data seem to indicate that a systematic antibiotic treatment is mandatory in the treatment of all *M. pneumoniae*-related neurological diseases. In early-onset cases with direct CNS damage, eradication of the infecting pathogen from the CSF seems essential to avoid its cytolytic activity and activation of the immune system with cytokine and chemokine production. In late-onset diseases, *M. pneumoniae* eradication from the respiratory tract can interrupt autoimmunity and related alterations. Macrolides are usually considered the drug of choice to treat *M. pneumoniae* infections for several reasons. First, these drugs have been used to treat these diseases with supposed or demonstrated *M. pneumoniae* etiology with excellent results for several decades (Waites and Talkington, 2004). Second, macrolides are generally well tolerated, safe and significantly less expensive than more recently developed drugs (Principi and Esposito, 1999). Third, macrolides have a strong immunomodulatory effect. They can enhance or reduce activation of the immune system through several different mechanisms, including regulation of the synthesis and/or secretion of proinflammatory and anti-inflammatory cytokines (Kwiatkowska and Maślińska, 2012). This effect leads to a significant down-regulation of the inflammation and to a positive influence on the outcome of several diseases in which abnormal activation of the immune system and inflammation play a relevant role in the development and persistence of clinical signs and symptoms (López-Boado and Rubin, 2008).

In the case of children, the subjects with the highest risk of *M. pneumoniae* infection, treatment with macrolides seems even more mandatory as other drugs that are theoretically active against *M. pneumoniae* cannot be used due to their several limitations. The use of tetracyclines in patients <8 years of age and chloramphenicol in the youngest of these can be followed by severe adverse events, and ketolides and streptogramins have limited used in pediatrics (Centers, 2018). Moreover, fluoroquinolones are not licensed for use in patients younger than 18 years of age with the exception of ciprofloxacin and levofloxacin, which are only recommended for a series of specific clinical conditions as urinary tract infections due to bacteria resistant to other antibiotics and respiratory exacerbations in patients with cystic fibrosis (Jackson and Schutze, 2016). However, the prescription of macrolides can be debated because of emergence of macrolide-resistat strains and the evidence that macrolides do not adequately cross the blood-brain barrier and do not reach effective concentrations in the CSF (Nau et al., 2010; Principi and Esposito, 2013; Pereyre et al., 2016). In neuropathies with direct-type damage, where the therapeutic target is the eradication of the pathogen from the CSF, the main limitation to the use of macrolides is the poor blood-brain barrier passage.

Treatment with alternative antibiotics that are fully effective against *M. pneumoniae*, such as tetracyclines and fluoroquinolones, has been shown to be effective in shortening the duration of respiratory symptoms and inducing rapid defervescence (Lee et al., 2018). Consequently, in cases with an indirect pathogenesis, there is a risk associated with the use of macrolides, particularly in those geographic areas where the prevalence of resistance is high. For these reasons, the choice of different antibiotics to treat neurological *M. pneumoniae* diseases seems mandatory. Although not licensed for use in children, quinolones, and in particular levofloxacin, seem the best solution. Levofloxacin adequately penetrates the CSF. Moreover, levofloxacin has been largely used off-label to treat children suffering from severe clinical conditions not treatable with or not responding to first-line antibiotics without any significant adverse event (Principi and Esposito, 2015). Evidence of the effective use of levofloxacin in *M. pneumoniae* neurologic diseases is given by the report by Esposito et al. (2011). These authors treated five patients with laboratory-confirmed *M. pneumoniae*-related meningoencephalitis (i.e., three with late-onset disease, one with early-onset disease and one with undetermined disease) with i.v. levofloxacin (25 mg/kg/day in two divided doses) for 14 days, showing a complete clinical recovery at the end of antibiotic administration without any significant adverse events. However, before levofloxacin can be considered the drug of choice for *M. pneumoniae* neurologic diseases, further data in a greater number of patients should be collected. The real efficacy of levofloxacin should be confirmed, and its drug dosage as well as therapy duration should be more precisely defined.

### Immunoglobulins and Anti-inflammatory and Immunosuppressive Drugs

Diseases characterized by acute severe inflammation and suspected or proved immunologic pathogenesis are usually treated with steroids with or without intravenous immunoglobulins (IVIG). Refractory cases receive plasma exchange (PE) and, if this measure is ineffective, immunosuppressive drugs or biologic response modifiers. Theoretically, all of these types of therapy can be used in *M. pneumoniae*-related neuropathies. However, adequate evaluation of their effectiveness is available only for diseases involving the PNS, mainly GBS syndrome. However, none of the studies consider the etiology of the GBS cases, and the real importance of treatment in *M. pneumoniae*-mediated cases is unknown. However, in GBS syndrome, corticosteroids are ineffective (Hughes et al., 2016). In contrast, both IVIG and PE have been found to be able to hasten the recovery in comparison to supportive treatment, particularly when treatment is initiated within 2 weeks of disease onset. No difference between treatments has been demonstrated in comparative studies. However, IVIG administration, due to its greater convenience and availability, is presently considered the first-line treatment for GBS and is recommended in severe cases. The combined use of IVIG and PE does not seem to offer real advantages, although the possibility of significant extra benefits could not be excluded (Hughes et al., 2014). A second IVIG course is suggested for patients who deteriorate in spite of initial therapy (Farcas et al., 1997). Few studies have evaluated the use of steroids, IVIG and immunosuppressive measures in *M. pneumoniae* neurologic disease with CNS involvement. Theoretically, these therapies could be, at least in part, effective because these treatments may promote the improvement or remission of many immune-mediated encephalopathies (80). On the other hand, with the progressive worsening of *M. pneumoniae*-associated encephalitis, steroids, IVIG and plasma exchange have been reported to be valid therapeutic options. The potential benefit of immune-modulating therapies is still unclear, but a reasonable consideration is their use in children with neurological symptoms leading to *M. pneumoniae*-induced autoimmune diseases (Principi et al., 2001).

## Conclusion

Several epidemiological and clinical studies have clearly indicated that *M. pneumoniae* can be associated with several PNS and CNS manifestations. The latency between a previous respiratory *M. pneumoniae* episode and the development of neurological signs and symptoms can be useful for distinguishing cases due to direct activity of the pathogen from those associated with an autoimmune mechanism. However, this differentiation is possible only in a minority of cases because a relevant number of neurologic diseases occurs without any recent history of respiratory problems. Moreover, even when *M. pneumoniae* infection is suspected, the identification of a recent episode of *M. pneumoniae* infection is frequently impossible because the signs and symptoms of the disease are not specific, and laboratory tests do not always offer adequate support. Finally, in cases with laboratory-confirmed *M. pneumoniae* infection, direct pathogenicity can be supported by the detection of *M. pneumoniae* DNA and/or inflammatory cytokines and chemokines such as IL-6 and IL-8 in the nervous system.

Unfortunately, evidence of positive tests using these measures is relatively uncommon due to the complexity of the laboratory tests. In addition to the long latency, an indirect pathogenesis can be supported by the presence of *M. pneumoniae* antibodies that cross-react with myelin components, although these components are difficult to identify and can also be detected, although more rarely, in subjects without neurological problems. The identification of cases with a vascular pathogenesis is even more complicated because, in these diseases, the precise causes of the damage have not been identified. The lack of knowledge of the pathogenesis of most of the cases of *M. pneumoniae*-mediated neurological diseases explains why treatment is not precisely defined. However, antibiotic treatment with drugs that are active against *M. pneumoniae* and able to pass the blood-brain barrier is recommended, even though the best drug, dosage, and duration of therapy have not been established. Moreover, some advances, such as the use of IVIG or PE, have been made for PNS *M. pneumoniae*-associated diseases, including GBS. Further studies should focus on the best therapy for *M. pneumoniae*-mediated neurological diseases.

## Author Contributions

RD wrote the first draft of the manuscript. EM, LG, and DL performed the literature review. NP co-wrote and supervised the manuscript. SE critically revised the paper. All authors read and approved the final version of the manuscript.

## Conflict of Interest Statement

The authors declare that the research was conducted in the absence of any commercial or financial relationships that could be construed as a potential conflict of interest.
